# Non-controlled, open-label clinical trial to assess the effectiveness of a dietetic food on pruritus and dermatologic scoring in atopic dogs

**DOI:** 10.1186/s12917-019-1929-2

**Published:** 2019-06-28

**Authors:** Angela Witzel-Rollins, Maryanne Murphy, Iveta Becvarova, Stephen R. Werre, Marie-Christine Cadiergues, Hein Meyer

**Affiliations:** 1 Department of Small Animal Clinical Sciences, The University of Tennessee College of Veterinary Medicine, 2407 River Drive, Knoxville, TN 37996 USA; 2 Hill’s Pet Nutrition Europe, 4106 Therwil, Grabetsmattweg, Switzerland; 3 Virginia-Maryland College of Veterinary Medicine, 205 Duck Pond Drive, Blacksburg, Virginia, 24061 USA; 4 UDEAR, Université de Toulouse, ENVT, INSERM, 23, Chemin des Capelles, 31076 Toulouse, cedex 3 France

**Keywords:** Canine, Atopy, Nutrition, Allergy, Dermatology, Veterinary, Diet

## Abstract

**Background:**

Canine atopic dermatitis (AD) is a common skin disease. The goal of this study was to evaluate food designed to improve skin barrier function and lower inflammation to reduce pruritus and clinical severity in client-owned atopic dogs. The food contained an antioxidant blend to reduce oxidative stress, plant polyphenols to stabilize mast cells, and polyunsaturated fatty acids to improve skin health and reduce inflammation.

**Results:**

Seventeen dogs were included in the analysis. Initially 48 adult atopic dogs were enrolled and exclusively fed a dermatologic food for 8 weeks in a non-controlled, open-label study. Thirty-one dogs were excluded for the following reasons: oral and topical medication changes (*n* = 17), missing data (*n* = 4), fatty acid supplementation (*n* = 3), food refusal (*n* = 3), dropped out (*n* = 3), and owner concerns (*n* = 1). Using a scale from 0 (normal) - 4 (severe), veterinarians evaluated the presence and severity of clinical signs of atopy at weeks 0, 4, and 8. Pet owners also rated their pet’s clinical signs of atopy on a scale from 0 (not present) - 10 (present continuously) at weeks 0, 4, and 8. Compared with initial baseline scores (median 19, range 3–69), the total veterinarian scores were significantly lower at weeks 4 (median 11, range 1–15) and 8 (median 7, range 3–46) (*p* < 0.05). Similarly, owner assessments showed significant improvements in the least squares mean (LSM) from baseline to 4 weeks (itching, redness, licking, and scratching) continuing to 8 weeks (itching, redness, and scratching) (*p* < 0.05).

**Conclusions:**

In this open, non-controlled study evaluating a dermatologic diet in seventeen client-owned dogs, owner and veterinarian assessments showed statistically significant reductions in clinical scores designed to measure severity of atopic dermatitis. While these results show promise for the management of canine atopic dermatitis, controlled clinical trials are also needed to affirm our findings.

**Electronic supplementary material:**

The online version of this article (10.1186/s12917-019-1929-2) contains supplementary material, which is available to authorized users.

## Background

Atopic dermatitis (AD) is a common disease of dogs and has been defined as pruritus and inflammation through immunoglobin E (IgE) production that predominately targets environmental antigens [1]. While the pathogenesis of AD is not completely understood, researchers have recently uncovered that AD involves the innate immune system and alterations in the epidermal barrier [2]. Traditional models of AD focused on genetic alterations, resulting in abnormal immune responses to typically harmless antigens. Recently, new theories contend that atopic patients have an abnormal skin barrier, which allows allergens and microbes to penetrate the epidermis and interact with immune cells. These models are not necessarily in opposition and it may be that a primary epidermal defect combines with an overactive immune response [3].

Treatment of AD is multifactorial and typically requires immune-modulation combined with allergen avoidance and strengthening of the epidermal barrier [4]. While the role of nutrition in canine AD needs more elucidation, a number of studies demonstrate that oral supplementation with polyunsaturated fatty acids (PUFA) improves pruritus and can lower the dosages of glucocorticoids and cyclosporine needed to control clinical signs [5–9]. The mechanisms of action for PUFAs effects on AD are still unclear, but likely involve a combination of reduced inflammatory cell activation, altered eicosanoid production, and improved epidermal barrier function [4, 10]. For example, incubation of peripheral blood polynuclear cells from atopic dogs with omega-3 fatty acids reduced cellular proliferation in vitro [11]. Additionally, a diet supplemented with the omega-3 fatty acids eicosapentanoic acid (EPA) and docosahexanoic acid (DHA) significantly lowered interleukin-1 and interleukin-6 activities and serum prostaglandin-E2 concentrations in healthy dogs [12]. Despite evidence suggesting omega-3 fatty acids reduce inflammation in normal dogs, fatty acid metabolism within skin tissue differs significantly from other organs and the effects of omega-3 fatty acids on inflammatory mediators of the skin in dogs with AD needs more investigation [4].

The intracellular lipid lamella of the stratum corneum (SC) is important for maintaining normal skin barrier function. The non-lesional skin of dogs with AD has reduced thickness and continuity of this lamellar layer [13, 14]. More specifically, lower ceramide levels have been demonstrated within the SC of AD dogs compared to normal controls [15, 16]. Oral supplementation of PUFAs appears to mitigate at least some of these structural changes [14].

Polyphenols are natural compounds found primarily in fruits and vegetables and contain an aromatic ring with multiple phenol groups attached [17]. There are several classes of polyphenols, including flavonoids, stilbenes, phenolic acids, and hydroxycinnamic acid lignans [18]. Examples of polyphenols include resveratrol, quercetin, epigallocatechin gallate (ECGC), and curcumin [18]. Polyphenols have been widely studied for their anti-neoplastic and anti-inflammatory properties [19–21].

There is also evidence to support the use of certain classes of polyphenols in allergic diseases [17, 18]. There are two main stages of the allergic immune response thought to be affected by polyphenols: allergen sensitization and allergen re-exposure [18]. Certain polyphenols can render antigenic proteins hypoallergenic by forming insoluble complexes [22]. Polyphenols can also affect antigen-presenting dendritic cells by interfering with their function and maturation [23, 24]. During allergen re-exposure, B cell antibody production and T cell cytokine production can also be reduced by polyphenols [18, 25–29]. In addition, quercetin has been shown to inhibit the release of inflammatory mediators from mast cells [30–32]. Orally administered quercetin and its derivatives (kaempferol, rutin) reduced the clinical signs and inflammation in a human clinical trial of patients with moderate to severe AD [33, 34]. Despite in vitro and in vivo evidence to support the use of polyphenols in allergic skin disease, research specifically targeting canine AD is still lacking.

Oxidative stress is an imbalance between pro-oxidant processes and antioxidant defenses and can disrupt cell signaling and arachidonic acid metabolism, resulting in systemic inflammation [35]. AD in both humans and dogs has been associated with higher oxidative stress [36, 37]. Vitamin C is an antioxidant important for maintaining skin health and humans with AD have significantly lower intradermal concentrations [38]. Plasma vitamin C concentrations also appear to inversely correlate with clinical severity of AD in people [39]. Vitamin E is another potent anti-oxidant that is particularly important for preventing lipid peroxidation. Some studies have shown dietary intake of vitamin E is inversely associated with allergic sensitization and serum IgE concentration in adult humans [40, 41]. In addition, randomized, placebo-controlled clinical trials in both dogs and humans have found reductions in clinical signs of AD with vitamin E supplementation [42, 43].

Management of AD should be multimodal. Adding nutritional intervention to commonly used therapies, such as immunomodulation, immunosuppression, and antimicrobial/antiparasitic medications, may further reduce pruritus and improve skin barrier functions. The goal of this study was to determine if a nutritionally complete and balanced diet containing high concentrations of PUFAs, vitamins with antioxidant activity, and polyphenols, such as quercetin, would improve the clinical signs and severity of AD in client owned dogs.

## Methods

All procedures were approved by the Hill’s Global Animal Welfare Committee and pet owners provided informed consent prior to participation. Client-owned dogs (the experimental units) of any breed with a history of environmental allergies, manifested by itching, licking, scratching, development of hot spots, and/or otitis due to atopy were recruited from 26 primary care and 2 dermatology veterinary facilities within 11 European countries. Consistency of diagnosis was enhanced via the presence of at least 5 satisfied criteria of atopic dermatitis.

Dogs could receive standard treatment for atopic dermatitis, including allergen avoidance, allergen-specific immunotherapy, symptomatic anti-inflammatory therapy (e.g., antihistamines, corticosteroids, antidepressants, cyclosporine, misoprostol, leukotriene inhibitors, phosphodiesterase inhibitors) and antimicrobial therapy (e.g., anti-fungal agents, antibiotics) as long as drugs, doses, and frequency of administration remained constant from the time of previous food administration through the completion of the study. Owners needed to be willing to feed the dermatologic diet exclusively for the duration of the trial. The nutrient composition and functional ingredients of the diet are located in Tables 1 and 2.^1^

**Table 1:** Nutrient composition of dermatologic diet

Nutrient	DMB	Per 1000 kcal
Crude protein	22.7%	55 g
Crude fat	17.0%	41 g
Carbohydrate	53.4%	130 g
Crude fiber	1.6%	4 g
Linoleic acid	4.59%	11.2 g
alpha-Linolenic acid	1.11%	2.7 g
Eicosapentaenoic + Docosahexaenoic acid	0.56%	1.38 g
Eicosapentaenoic acid	0.33%	0.81 g
Docosahexaenoic acid	0.23%	0.57 g
Total n-6 fatty acids	4.76%	11.6 g
Total n-3 fatty acids	1.81%	4.42 g
Zinc	267 mg/kg	0.065 g
Vitamin A	10,638 IU/kg	259 IU
Vitamin E	874 mg/kg	0.21 g
Vitamin C	98 mg/kg	0.024 g
Beta carotene	1.6 mg/kg	0.4 mg
Energy (kcal)	411/kg	376/kg as fed
Energy (kJ)	1720/kg	1573/kg

Legend: Nutrient composition of the dermatologic diet on a dry matter basis (DMB) and per 1000 kcal basis

**Table 2:** Classification of major functional dietary ingredients in test diet

Polyunsaturated Fatty Acids	Polyphenol	Antioxidant
Soybean oil	Flaxseed	Green tea
Flaxseed	Brewer’s rice	Brewer’s rice
Fish oil	Rosemary	Dried beet pulp
	Green tea	Rosemary
	Citrus pulp	Vitamin E
		L-Ascorbyl-2-Polyphosphate (source of Vitamin C)
		Beta-carotene

Legend: Classification of functional dietary ingredients in test diet^a^ [44–48]

Dogs were excluded if they had untreated cases of parasitic or infectious dermatitis or were diagnosed with concurrent skin conditions (e.g., flea bite allergic dermatitis, sarcoptic mange, Cheyletiella, Demodex canis, Trombicula autumnalis, Trichodectes canis, and/or acute flare-ups of bacterial pyoderma or Malassezia). In addition, dogs had to be > 1 year of age and could not be pregnant or nursing. Dogs with major concurrent systemic diseases, such as diabetes mellitus, hypothyroidism, hyperadrenocorticism, and chronic kidney disease, as well as dogs with anticipated or planned surgery during the feeding period were also excluded. Any dog consuming fish oil supplements or foods known to contain high levels of EPA and DHA (> 500 mg/1000 kcal of EPA and DHA combined) within 12 weeks before the start of the study were not enrolled. An unwillingness of dog owners to feed the test diet exclusively for 8 weeks, discontinue homeopathic, nutraceutical, vitamin or nutritional supplements (e.g., vitamin E, fatty acid supplements, glucosamine/chondroitin sulfate, antioxidants), and planned use of oclacitinib (Apoquel®) also served as exclusion criteria.^2^

The present study was a non-controlled, open-label, 8-week long feeding survey. Dogs were treated for parasites and infections prior to official enrollment and initiation of the feeding trial. Dogs were seen at three separate visits: visit 1 (recruitment), visit 2 (4 weeks after starting the dermatologic diet), and visit 3 (8 weeks after starting the dermatologic diet). The presence of Favrot’s criteria for canine atopic dermatitis was evaluated at visit 1 (recruitment). Dog owners received the dermatologic diet along with instructions regarding recommended daily feeding quantities. Owners were instructed to exclusively feed the dermatologic diet and discontinue homeopathic, nutraceutical, and vitamin or nutritional supplements. Owners were encouraged to avoid treats during the trial, but a portion of the daily-allocated dermatologic diet kibble could be given as needed during the day as a treat source. At each visit, the attending veterinarian completed a dermatological evaluation based on the modified Canine Atopic Dermatitis Extent and Severity Index (CADESI-03) [49] (Additional file 1: Figure S1). Dogs were evaluated using the clinical assessment for 12 body sites (1. Chin, lips & face, 2. Concave pinnae, 3. Axillae, 4. Front paws, 5. Hind paws, 6. Cubital flexor, 7. Palmar metacarpal area, 8. Dorsum, flank & tail base, 9. Inguinal area, 10. Abdomen, 11. Perineum, 12. Ventral tail) using lesion images. Each body site was evaluated for the degree of erythema, lichenification, excoriations and alopecia using the following scoring system: 0 = None, 1 = Very mild, 2 = Mild, 3 = Moderate, 4 = Severe (Additional file 1: Figure S1). Body weight, body condition score (BCS, 1–5/5) [50], and changes to the treatment protocol were recorded at each visit. Owners completed a subjective evaluation form at each visit using a visual analog scale, which included information regarding their opinion of their dog’s quality of life, skin and coat quality, and acceptance of the dermatologic diet (Additional file 2: Figure S2). At visit 3, veterinarians recorded their opinion regarding effectiveness of the diet for helping manage atopic dermatitis, ease of use of the diet compared to other dietary options, and likelihood of diet recommendation. During and after completion of the study, dogs remained in their owner’s homes under the supervision of their veterinarians.

Normal probability plots showed that veterinarian dermatological scores (total score over the entire dog and for each of the 12 regions within the dog) and body condition scores were skewed. Normal probability plots also showed that body weight and all of the owner scores were normally distributed. Accordingly, data were summarized as medians with a range (veterinarian dermatological scores and body condition scores) and least square means with a standard error (body weight and owner assessment scores).

Null Hypotheses were three fold: 1) The new diet will not have an effect on the dermatological scores recorded by veterinarians over time (statistically, the median scores at baseline will be equal to the median score at 4 weeks which also be equal to the median score at 8 weeks). 2) The new diet will not have an effect on owner assessment scores recorded over time (statistically, the least squares means at baseline will be equal to the least squares means at 4 weeks which also will be equal to the least squares means at 8 weeks). 3) The new diet will not have an effect on body weight recorded over time (statistically, the least squares means at baseline will be equal to the least squares means at 4 weeks which also will be equal to the least squares means at 8 weeks). 4) The new diet will not have an effect on body condition scores recorded over time (statistically, the median scores at baseline will be equal to the median score at 4 weeks which also be equal to the median score at 8 weeks). To test hypotheses 1 and 4, dermatological scores recorded by veterinarians (modified CADESI) and body condition scores (separately) were compared between time points (baseline vs. 4 weeks vs. 8 weeks) using Friedman’s chi-square test with dog identification as a blocking factor. *P*-values for the 2-way comparisons were adjusted for multiple comparisons using Bonferroni’s procedure. To test hypotheses 2 and 3, owner assessment scores and body weight were compared between time points using mixed-model repeated-measure analysis of variance followed by Tukey’s procedure for multiple comparisons. The general linear mixed model specified weeks at follow-up as a fixed effect with the Kenward-Roger approximation as the denominator degrees of freedom. G-side variation in the data was modeled by specifying dog identification as a random effect, while the R-side variation in the data was modeled by specifying a first order autoregressive covariance matrix.

Statistical significance was set at α = 0.05. All analyses were performed using SAS version 9.4 (Cary, NC, USA).

## Results

Seventeen dogs were included in the analysis. Initially, 48 adult dogs with AD were enrolled.. Thirty-one dogs were excluded for the following reasons: oral medication changes (*n* = 14), initiation of topical antimicrobial shampoo (*n* = 3), missing data (*n* = 4), fatty acid supplementation (n = 3), food refusal (n = 3), dropped out (n = 3), and owner concerns (n = 1). Eight of the 14 dogs with oral medication changes stopped the use of antimicrobials (n = 4) and/or immunosuppressive drugs (*n* = 5). The remaining dogs with oral drug changes started medications, such as cyclosporine (*n* = 2), prednisone (n = 1), oclacitinib (n = 3), dexamethasone (n = 1), or immunotherapy (n = 1). One dog stopped oclacitinib and started dexamethasone. Within the set of dogs analyzed, there were missing pet owner assessment scores, so samples sizes for individual assessments ranged from 7 to 16. Demographics of the analyzed patients can be found in Table 3. Patients ranged from 2 to 13 years of age with a mean of 6.1 years and included 10 spayed females, 2 intact females, 2 neutered males, and 3 intact males. Initial enrollment dates for patients included in the final analysis ranged from January 28, 2016 to June 24, 2016.

**Table 3:** Characteristics of dogs included in data analysis

Dog ID	Country	Enrollment date	Breed	Sex	Age in years
6	Belgium	5/4/16	French Bulldog	NM	3
11	France	2/29/16	Shih Tzu	NF	11
12	France	5/26/16	Lhasa apso	F	9
16	France	6/24/16	French Bulldog	NF	6
19	France	1/28/16	Jack Russel Terrier	NF	3
20	France	5/23/16	Cavalier King Charles	NF	3
22	France	2/12/16	French Bulldog	NF	n/a
23	France	5/20/16	Labrador	NF	2
25	France	2/11/16	Newfoundland x Labrador Mix	M	10
26	France	4/18/16	Shih Tzu	NM	13
27	France	3/15/16	Boxer	F	3
28	Germany	5/19/16	Labrador	NF	7
31	Greece	3/2/16	Mongrel	NF	10
33	Italy	3/10/16	Wirehaired Dachshund	M	2
34	Italy	4/26/16	Shiba Inu	NF	7
35	Italy	4/11/16	American Staffordshire	NF	2
37	Lithuania	n/a	Cocker Spaniel	M	7

Legend: Demographics of animals included in final study analysis. F = female, NF = neutered female, M = male, NM = neutered male

**Fig. 1:**
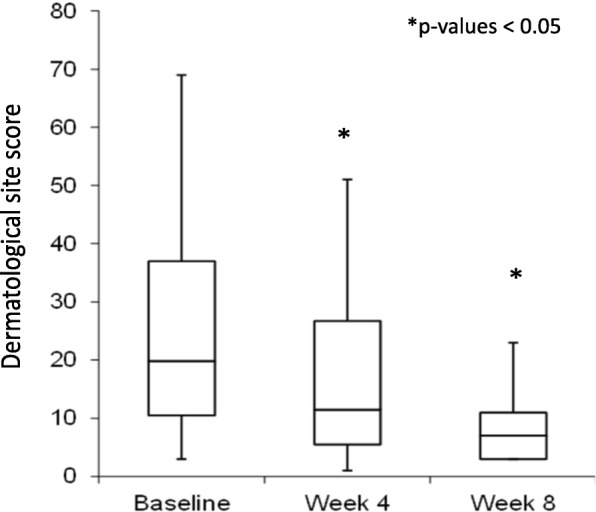
Total dermatological site score assessments by veterinarians. Dermatologic scores for all regions were combined into total dermatologic site score. Subjective assessments (normal/none = 0, very mild = 1, mild = 2, moderate = 3 and severe = 4) of erythema, lichenification, excoriations and alopecia at 12 body sites were combined to give a total score of 0–192. Median total dermatological site score assessed by veterinarians was significantly lower at 4 weeks and at 8 weeks (*p < 0.05) when compared with baseline

The median total dermatological score (all regional sites combined) from veterinarians (modified CADESI) was significantly lower at 4 weeks (median 11, range 1–15) and at 8 weeks (median 7, range 3–46) when compared with the baseline (median 19, range 3–69) (*p* values < 0.05, Fig. 1, Table 4). By the end of the study, median overall scores were reduced by 63% compared to baseline (19 to 7). The majority of improvement was seen within 4 weeks with median total scores decreasing 42% (19 to 11). Improvement continued from week 4 to 8 with median scores decreasing another 37% (11 to 7).

**Table 4:** Modified Canine Atopic Dermatitis Extent and Severity Index scores recorded by veterinarians during the study period

Dermatological site	Visit			*P*-value
	Initial	4 weeks	8 weeks	
Dog total	19 (3–69)^a^	11 (1–51)^b^	7 (3–46)^bc^	0.0001
Chin, Lips & Face (left and right combined)	3 (0–8)^a^	1 (0–6)^b^	0 (0–5)^bc^	0.0003
Medial Pinnae (concave pinnae)	2 (0–11)	2 (0–9)	1 (0–5)	0.1046
Axillae	2 (0–10)^a^	0 (0–6)^ab^	0 (0–4)^b^	0.0020
Front Paws (dorsal and palmar sides combined)	3 (0–7)^a^	1 (0–4)^b^	1 (0–4)^ab^	0.0017
Hind Paws (dorsal and plantar sides combined)	3 (0–7)	1 (0–7)	1 (0–10)	0.2224
Cubital Flexor (elbow folds)	0 (0–13)	0 (0–4)	0 (0–4)	0.0490
Palmar Metacarpal (from carpal to metacarpal pads)	0 (0–4)	0 (0–4)	0 (0–4)	0.1673
Dorsum, Flanks & Tail Base	0 (0–11)^a^	0 (0–6)^a^	0 (0–2)^a^	0.0383
Inguinal Areas†	1 (0–10)^a^	1 (0–6)^b^	0 (0–4)^ab^	0.0337
Abdomen	1 (0–10)	1 (0–8)	0 (0–4)	0.1619
Perineum	0 (0–9)	0 (0–6)	0 (0–4)	0.1058
Ventral Tail (proximal)	0 (0–8)	0 (0–4)	0 (0–4)	0.1353

Legend: Modified Canine Atopic Dermatitis Extent and Severity Index scores recorded by veterinarians during the study period. Data are reported as median (range). Data were available for 17 dogs during the initial examination and at 4 weeks, and for 13 dogs at 8 weeks

^a,b,c^ Within each row, time points with different letters are significantly different (Friedman’s chi-square with Bonferroni adjustment < 0.05)

^†^ Statistical significance is not robust for this region (Statistical test looks at the entire distribution. For borderline cases, it is possible to have statistical significance with equal medians)

There were also significant regional improvement in modified CADESI scores over time. Areas around the face (medians scores [range] at baseline, 4 weeks and 8 weeks were 3 [0–8], 1 [0–6], 0 [0–5], respectively), axillary (medians scores [range] at baseline, 4 weeks and 8 weeks were 2 [0–10], 0 [0–6], 0 [0–4], respectively), and front paws (medians scores [range] at baseline, 4 weeks and 8 weeks were 3 [0–7], 1 [0–4], 1 [0–4], respectively) had the most significant improvements (*p* values < 0.005) (Table 4). The flank, dorsum, and tail region (medians scores [range] at baseline, 4 weeks and 8 weeks were 0 [0–11], 0 [0–6], 0 [0–2], respectively) also had statistically significant score reductions (*p* < 0.05).

**Fig. 2:**
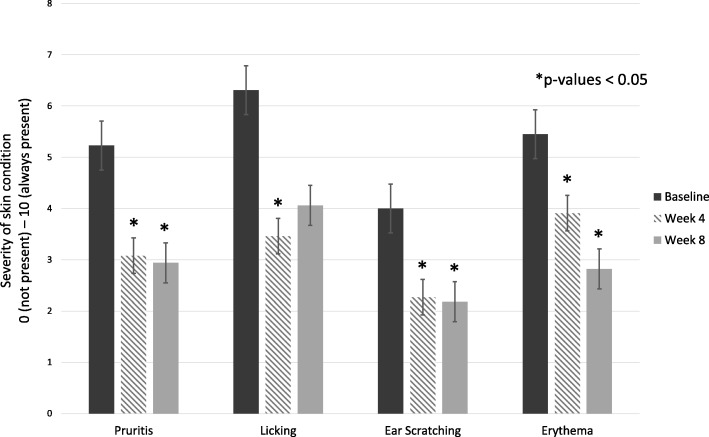
Conditions showing significant improvement based on dog owner assessments. Owner assessments of skin condition showed significant improvements in least squared mean (LSM) at 4 and 8 weeks (*p < 0.05) when compared with baseline

Compared with baseline observations, owner assessments showed significant improvements in the least squares mean (LSM) of pruritus/scratching, erythema and licking from baseline to 4 weeks (pruritus/scratching, erythema, and licking) and on to 8 weeks (pruritus/scratching and erythema) (p < 0.05, Fig. 2) in the 17 dogs completing the study. At week 4, licking, erythema, and pruritus/scratching scores decreased by 45% (6.31 to 3.46), 28% (5.45 to 3.91), and 41% (5.23 to 3.08), respectively. By week 8, erythema scores were reduced by 48% (5.45 to 2.82) and pruritus/scratching scores were reduced by 44% (5.23 to 2.94) compared to baseline (Table 5).

**Table 5:** Quality of life and skin condition as perceived by dog owners during the study period

Parameter rated	LSM (SE)/N*	Visit			*P*-value
		Week 0	Week 4	Week 8	
Acceptance	LSM (SE)	5.00 (0.19)	4.60 (0.19)	4.60 (0.19)	0.1408
	N	5	5	5	
Dandruff	LSM (SE)	2.82 (0.69)	1.09 (0.69)	1.91 (0.69)	0.0743
	N	11	11	11	
Disruption to family	LSM (SE)	3.18 (0.93)	2.36 (0.93)	2.36 (0.93)	0.0967
	N	11	11	11	
Hair loss	LSM (SE)	5.91 (0.99)	4.27 (0.99)	4.55 (0.99)	0.0684
	N	11	11	11	
Head shaking	LSM (SE)	3.15 (0.82)	2.31 (0.82)	2.26 (0.84)	0.1617
	N	13	13	11	
Licking	LSM (SE)	6.31 (0.67)^a^	3.46 (0.67)^b^	4.06 (0.73)^ab^	0.0055
	N	13	13	11	
Odor	LSM (SE)	2.45 (0.74)	1.73 (0.74)	1.27 (0.74)	0.0521
	N	11	11	11	
Overall condition of skin	LSM (SE)	4.64 (0.78)	3.45 (0.78)	3.27 (0.78)	0.1332
	N	11	11	11	
Quality of life	LSM (SE)	6.86 (0.68)	6.93 (0.68)	7.51 (0.73)	0.6296
	N	14	14	11	
Redness/erythema	LSM (SE)	5.45 (0.82)^a^	3.91 (0.82)^b^	2.82 (0.82)^c^	0.0007
	N	11	11	11	
Rubbing	LSM (SE)	4.85 (0.83)	4.54 (0.83)	3.82 (0.89)	0.6333
	N	13	13	11	
Scratching all skin	LSM (SE)	5.23 (0.87)^a^	3.08 (0.87)^b^	2.94 (0.88)^bc^	0.0001
	N	13	13	11	
Scratching of ears	LSM (SE)	4.00 (0.87)	2.27 (0.87)	2.18 (0.87)	0.0129
	N	11	11	11	
Shininess of hair	LSM (SE)	3.73 (0.80)	2.73 (0.80)	3.55 (0.80)	0.2195
	N	11	11	11	
Softness of hair	LSM (SE)	3.64 (0.75)	2.91 (0.75)	3.27 (0.75)	0.6753
	N	11	11	11	

Legend: Dog owner assessment scores of various quality of life and atopic dermatitis clinical signs at baseline, week 4, and week 8. *LSM = least squares mean; SE = Standard error; N = number of dogs for which data were available at each time point. ^a,b,c^ Within each row, time points with different letters are significantly different (Tukey’s *p*-value < 0.05)

Median BCS at the start of the study was 3.2 (3–4) out of 5 and did not significantly change (*p* = 0.61). Mean initial body weight was 17.3 kg (SE 3.18) and did not significantly change during the course of the study (*p* = 0.45).

## Discussion

In this open-label, uncontrolled clinical trial, dogs with AD showed marked improvements from both pet owners and veterinarians when fed a diet high in PUFAs, antioxidants, and polyphenols. The concentration of EPA and DHA in the test diet were comparable to dosages used in other clinical trials of canine skin disease (1.35 g/1000 kcal) when dogs met daily energy requirements as estimated by the National Research Council (95–180 × kg ^0.75^) [5, 6, 51]. Since previous studies have demonstrated improvement with omega 3 fatty acid supplementation alone, it is difficult to determine the role of other active ingredients in the trial diet. In particular, polyphenols have not been singularly evaluated for mitigation of canine atopic signs.

Atopic dermatitis can severely affect a pet’s quality of life and is of high concern for most pet owners. Health-related quality of life (HRQoL) refers to the subjective perception of the impact of health status on life quality. Specific HRQoL domains that are represented by the improvements include social environment, physical health (i.e., itching, redness, overall skin condition), and self-care (i.e., licking) [52]. Seventy-three percent of owners of dogs with AD consider AD as having a major impact on their dog’s HRQoL and the perception is affected by the extent of pruritus and CADESI [53]. A third of owners also consider treatment to be a major burden for their dog [53]. The significant improvements in the LSM scores of itching, redness, licking, and scratching found in this study may indicate the dogs developed an improved HRQoL while consuming the trial diet. Owners in the current study were asked to judge improvement in their pet’s quality of life (QoL) over the prior 7 days at all 3 visits. While owner perception of QoL did not change over the 8-week period, owners did perceive improvements in criteria used to determine HRQoL. Therefore, it is reasonable to infer that the improvements in itching and modified CADESI found with the test diet in this study may contribute to improved HRQoL in dogs with AD. Owner education regarding these improvements may also improve their perception of general QoL.

Overall acceptance of the test diet was good, with almost 90% of dogs initially enrolled eating the diet for the entire eight-week period and mean body condition score remaining unchanged. A large proportion of dogs initially enrolled in the study had to be removed due to changes in oral medications. The number of dogs that started and stopped medications was almost even, thus removing selection bias towards more or less severe cases.

Being open-label without a control group and a small sample size are major limitations of this study. Pet owners and veterinarians may have scored dogs more favorably knowing they were receiving a diet designed to improve AD. In addition, AD is affected by seasonal changes in pollens, grasses, and other environmental allergens. Most patients were initially enrolled in the 2 month study during late winter (*n* = 7) and spring (*n* = 8) when seasonal allergies are often worsening. Therefore, seasonal allergies would be more likely to reduce diet benefits than to enhance them. However, the addition of a control diet group would be required to accurately determine seasonal effects on the study. Additionally, extending the timeline of the trial to an entire year or performing trials in different seasons could help negate seasonal effects.

While the scale used for dermatologic evaluation in this study was based on the CADESI-03 scale, it was modified from the original. The objectives of the modifications were to preserve the validated parameters (types of body sites), simplify the protocol, and obtain objective, consistent and intra- and inter-observer reliable data. While this scale was not validated, it was based on the same principles as CADESI scale. Furthermore, it was used consistently in all enrolled patients, thus it allowed objective evaluation of the differences from the baseline to week 4 and week 8. A major limitation of our scale is that our data cannot be compared head to head with other studies.

This uncontrolled, open-label clinical trial demonstrates that significant clinical improvement as assessed by both pet owners and veterinarians can be achieved by feeding a diet designed to support skin barrier function, while also reducing inflammation and oxidative damage. Utilizing diet to manage AD is a safe and easy therapeutic strategy for pet owners that may complement other treatments. While feeding the test diet alone did not result in complete resolution of clinical signs, it may allow clinicians to reduce dosages or the number of medications prescribed for AD. Our results suggest significant improvement in clinical signs of AD should be seen within 4 weeks of feeding the test diet, with improvement continuing through 8 weeks. Whether dogs would continue to improve past 8 weeks was not evaluated. While more research is needed to confirm the results of this study and determine which dietary compounds are providing the most beneficial effect, the ingredients and formulation of this test diet hold promise for AD management.

## Conclusions

In an open-label, uncontrolled clinical trial, 17 dogs with atopic dermatitis demonstrated marked improvement from both pet owners and veterinarians when fed a dermatologic diet.

## Additional files

Additional file 1: **Figure S1.** Dermatological evaluation chart provided to veterinarians. Veterinarians were asked to fill in the table above to describe skin lesions and body regions affected at weeks 0, 4, and 8. (DOCX 73 kb)

Additional file 2: **Figure S2.** Dog owner assessment form. Dog owners were asked to complete the assessment form to rate their dog’s quality of life, skin and coat quality, and acceptance of the dermatologic diet at weeks 0, 4, 8. (DOCX 13 kb)

## Data Availability

All data generated or analyzed during this study are included in the supplementary information files.

## Abbreviations

AD: Atopic dermatitis
DHA: Docosahexaenoic acid
ECGC: Epigallocatechin gallate
EPA: Eicosapentaenoic acid
IgE: Immunoglobulin E
PUFA: Polyunsaturated fatty acid
SC: Stratum corneum

## Footnotes

1 Hill’s® Prescription Diet® Derm Defense™ Canine, Hills Pet Nutrition, Topeka, KS

2 Apoquel®, Zoetis, Parsippany, New Jersey
