# Supplementary material for: Non-controlled, open-label clinical trial to assess the effectiveness of a dietetic food on pruritus and dermatologic scoring in atopic dogs
Source: BMC Vet Res. 2019 Jun 28;15:220. doi: 10.1186/s12917-019-1929-2 (PMC6599232; doi:10.1186/s12917-019-1929-2)
Supplement: Supplementary file 1 — Figure S1. Dermatological evaluation chart provided to veterinarians. Veterinarians were asked to fill in the table above to describe skin lesions and body regions affected at weeks 0, 4, and 8. (DOCX 73 kb) [file 12917_2019_1929_MOESM1_ESM.docx]

| **Dermatological evaluation**  Please provide a clinical assessment for each of the numbered sites, using the lesion images and using the following scoring system: **0 = None, 1 = Very Mild, 2 = Mild, 3 = Moderate, 4 = Severe**   \| 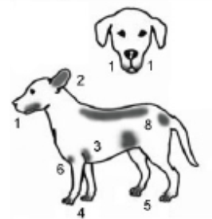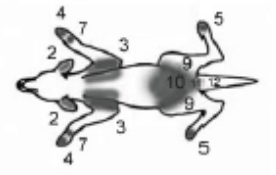 \| **Erythema** \| **Lichenification** \| **Excoriations** \| **Alopecia** \| \| --- \| --- \| --- \| --- \| --- \| \| **0-4** \| **0-4** \| **0-4** \| **0-4** \| \| 1. Chin, Lips & Face (left and right combined) \|  \|  \|  \|  \| \| 2. Medial Pinnae (concave pinnae) \|  \|  \|  \|  \| \| 3. Axillae \|  \|  \|  \|  \| \| 4. Front Paws (dorsal and palmar sides combined) \|  \|  \|  \|  \| \| 5. Hind Paws (dorsal and plantar sides combined) \|  \|  \|  \|  \| \| 6. Cubital Flexor (elbow folds) \|  \|  \|  \|  \| \| 7. Palmar Metacarpal (from carpal to metacarpal pads) \|  \|  \|  \|  \| \| 8. Dorsum, Flanks & Tail Base \|  \|  \|  \|  \| \| 9. Inguinal Areas (groin) \|  \|  \|  \|  \| \| 10. Abdomen \|  \|  \|  \|  \| \| 11. Perineum (from vulva/scrotum to anus) \|  \|  \|  \|  \| \| 12. Ventral Tail (proximal) \|  \|  \|  \|  \| |
| --- | --- | --- | --- | --- | --- | --- | --- | --- | --- | --- | --- | --- | --- | --- | --- | --- | --- | --- | --- | --- | --- | --- | --- | --- | --- | --- | --- | --- | --- | --- | --- | --- | --- | --- | --- | --- | --- | --- | --- | --- | --- | --- | --- | --- | --- | --- | --- | --- | --- | --- | --- | --- | --- | --- | --- | --- | --- | --- | --- | --- | --- | --- | --- | --- | --- | --- | --- | --- | --- |
