# Supplementary material for: Non-controlled, open-label clinical trial to assess the effectiveness of a dietetic food on pruritus and dermatologic scoring in atopic dogs
Source: BMC Vet Res. 2019 Jun 28;15:220. doi: 10.1186/s12917-019-1929-2 (PMC6599232; doi:10.1186/s12917-019-1929-2)
Supplement: Supplementary file 2 — Figure S2. Dog owner assessment form. Dog owners were asked to complete the assessment form to rate their dog’s quality of life, skin and coat quality, and acceptance of the dermatologic diet at weeks 0, 4, 8. (DOCX 13 kb) [file 12917_2019_1929_MOESM2_ESM.docx]

| How would you rate your dog’s quality of life over the **last 7 days** on a scale from **0 (very poor) to 10 (very high):**  0 1 2 3 4 **5** 6 7 8 9 10 |
| --- |
| **Thinking of your dog’s skin & coat, please rate the following:**  **HOW MUCH RUBBING OF HIS/HER FACE THAT YOUR DOG APPEARS TO BE DISPLAYING** on a scale from **0 (not rubbing at all) to 10 (rubbing all of the time):**  0 1 2 3 4 **5** 6 7 8 9 10  **THE AMOUNT OF SCRATCHING/ITCHING OF HIS/HER SKIN THAT YOUR DOG IS CURRENTLY DISPLAYING** on a scale from **0 (not scratching at all) to 10 (scratching all of the time):**  0 1 2 3 4 **5** 6 7 8 9 10  **THE AMOUNT OF LICKING OF HIS/HER PAWS, ARMPITS, GROIN, AND/OR ANAL AREA THAT YOUR DOG IS CURRENTLY DISPLAYING** on a scale from **0 (not licking at all) to 10 (licking all of the time):**  0 1 2 3 4 **5** 6 7 8 9 10  **HOW MUCH HEAD SHAKING THAT YOUR DOG APPEARS TO BE DISPLAYING** on a scale from **0 (not shaking at all) to 10 (shaking all of the time):**  0 1 2 3 4 **5** 6 7 8 9 10  **HOW MUCH SCRATCHING OF HIS/HER EARS THAT YOUR DOG APPEARS TO BE DISPLAYING** on a scale from **0 (not scratching at all) to 10 (scratching all of the time):**  0 1 2 3 4 **5** 6 7 8 9 10  **HOW DISRUPTIVE YOUR DOG’S SKIN CONDITION IS TO YOU OR YOUR FAMILY** on a scale from **0 (not disruptive at all) to 10 (extremely disruptive):**  0 1 2 3 4 **5** 6 7 8 9 10  **THE REDNESS OF YOUR DOG’S SKIN IF YOUR DOG IS CURRENTLY DISPLAYING RED AREAS** on a scale from **0 (not red at all) to 10 (extremely red):**  0 1 2 3 4 **5** 6 7 8 9 10  **THE AMOUNT OF ODOR YOUR DOG’S SKIN CONDITION IS CAUSING** on a scale from **0 (no odor at all) to 10 (extremely smelly):**  0 1 2 3 4 **5** 6 7 8 9 10 |
| **THE CURRENT OVERALL CONDITION OF YOUR DOG’S SKIN AND HAIRCOAT** on a scale from **0 (very healthy) to 10 (extremely poor):**  0 1 2 3 4 **5** 6 7 8 9 10  **THE SHININESS OF YOUR DOG’S HAIR** on a scale from **0 (very shiny) to 10 (very dull):**  0 1 2 3 4 **5** 6 7 8 9 10  **THE SOFTNESS OF YOUR DOG’S HAIR** on a scale from **0 (very soft) to 10 (very coarse or brittle):**  0 1 2 3 4 **5** 6 7 8 9 10  **HOW MUCH HAIR LOSS THAT YOUR DOG APPEARS TO BE CURRENTLY DISPLAYING** on a scale from **0 (no hair loss) to 10 (extreme hair loss):**  0 1 2 3 4 **5** 6 7 8 9 10  **HOW MUCH DANDRUFF YOUR DOG APPEARS TO BE CURRENTLY DISPLAYING** on a scale **from 0 (no dandruff at all) to 10 (extremely scaly scurf):**  0 1 2 3 4 **5** 6 7 8 9 10 |
| How would you rate your dogs' acceptance/eating enjoyment of the Derm Defense food?  1 poor 2 fair 3 average 4 very good 5 excellent |
